# Secondary antibody deficiencies in the modern era: emerging trends, diagnostic pitfalls, and advances in personalised management

**DOI:** 10.3389/fimmu.2025.1635094

**Published:** 2025-10-07

**Authors:** Shuayb Elkhalifa, Fulvio Salvo, Haggar Elbashir, Irfan Shafiq, Saed Isse, Mohamed Abuzakouk, Mohamed Medhat Gaber, Rehan Bhana

**Affiliations:** ^1^ Cleveland Clinic Abu Dhabi, Abu Dhabi, United Arab Emirates; ^2^ University of Manchester, Manchester, United Kingdom; ^3^ Lancashire Teaching Hospitals National Health Service (NHS) Foundation Trust, Preston, United Kingdom; ^4^ Birmingham City University, Birmingham, United Kingdom

**Keywords:** secondary antibody deficiencies, hypogammaglobulinaemia, immunoglobulin replacement therapy, recurrent infections, haematological malignancy, immunosuppressive therapy, artificial intelligence, electronic health records

## Abstract

Secondary antibody deficiencies (SADs) are a significant but frequently under-recognised group of acquired immunodeficiencies. They may arise in various clinical settings, including haematological malignancies, immunosuppressive therapies, and protein-losing conditions. SADs are associated with an increased risk of recurrent and severe infections, hospitalisation, and impaired quality of life. Despite this, diagnostic and treatment pathways remain inconsistent across healthcare settings and regions. Recent advances in the use of structured clinical data, including electronic health records and systematic laboratory assessments, show promise in facilitating earlier recognition of SADs. These approaches support more timely treatment decisions and promote consistent standards of care. Achieving improved outcomes for individuals with SADs will require broader consensus on diagnostic criteria, treatment thresholds, and access to specialist immunology services.

## 1 Introduction

Secondary antibody deficiencies (SADs) are acquired immunodeficiencies characterised by reduced levels or impaired function of immunoglobulins. Unlike primary antibody deficiencies (PADs), which arise from intrinsic genetic defects, SADs result from external or systemic factors such as malignancy, immunosuppressive treatment, or protein-losing conditions (1, 2) among other factors (3–6).

With regards to the prevalence, PADs have been estimated to be around 1 in 1,200 individuals of any age (3); in contrast, SADs are estimated to be 30-fold more common than PADs (1, 2). SADs have become increasingly relevant due to the expanding use of immunosuppressive and biological therapies, solid organ transplantation, and a range of other acquired causes such as malnutrition, increased catabolism, or iatrogenic losses including plasma exchange and peritoneal dialysis, among others described below (2–6).

SADs encompass a broad spectrum of underlying causes. Common settings include haematological malignancies, such as chronic lymphocytic leukaemia (CLL) and multiple myeloma (MM), where progressive disease or therapy can disrupt normal B-cell function and immunoglobulin production (1, 2, 7). Protein-losing conditions (6), including nephrotic syndrome and protein-losing enteropathies, can lead to significant immunoglobulin loss even in the presence of normal production (2, 6). Severe malnutrition and critical illness can further contribute to impaired immunoglobulin synthesis or increased catabolism (2, 6).

The clinical burden of SAD is significant and includes an increased risk of recurrent and often severe infections (1, 2, 8). These infections typically affect the sinopulmonary and gastrointestinal tracts and can result in complications such as bronchiectasis, hospitalisation, and prolonged antibiotic exposure (8). Infections with encapsulated bacteria such as *Streptococcus pneumoniae* and *Haemophilus influenzae* are particularly common (1, 8). Beyond infections, SAD may also contribute to impaired responses to vaccination (1, 2), difficulties in managing the underlying disease, and reduced quality of life for affected individuals (9).

Despite these challenges, SADs are often underdiagnosed or identified late (1, 2, 8, 9). Testing for immunoglobulin levels and functional antibody responses is not consistently incorporated into routine care (10), and thresholds for initiating treatment with immunoglobulin replacement therapy (IgRT) vary between regions and specialties (10, 11). Current evidence indicates that early identification of SAD and appropriate treatment (with either IgRT (11) or antimicrobial prophylaxis (7, 8)) can help to reduce infection burden and improve clinical outcomes (7, 8, 11). Treatment decisions should be based not solely on IgG cut-offs, but also on infection burden (8), functional antibody assessment (10, 11), and longitudinal follow-up (10, 11).

Efforts to close these gaps have highlighted the importance of thorough clinical review and data interpretation (9). Structured clinical data and systematic assessment of immunological markers, such as immunoglobulin levels (9) and vaccine responses (9), are proving valuable in identifying patients at risk of SAD (8, 10). These tools support the timely evaluation of individuals receiving immunosuppressive therapy and those with recurrent infections, promoting more consistent and effective care (7, 10).

The aim of this narrative review is to provide clinicians with an overview of SADs, and exploring the evolving diagnostic challenges, treatment strategies, and the role of digital health tools. Sources were identified via PubMed searches of peer-reviewed English-language articles from 2000 to 2025 using combinations of keywords including ‘secondary immunodeficiency,’ ‘hypogammaglobulinaemia,’ and ‘immunoglobulin replacement therapy’.

## 2 Classification and causes of secondary antibody deficiencies

SADs can be broadly classified according to the underlying mechanism: reduced production of immunoglobulins (12), increased loss of immunoglobulins (12), or a combination of both (2–4, 9, 12). Understanding these mechanisms is essential for accurate diagnosis and appropriate treatment.

### 2.1 Impaired antibody production

Reduced immunoglobulin production is a frequent cause of SADs, particularly in the context of haematological malignancies such as CLL (5,) MM (7), and non-Hodgkin lymphoma (5, 8).In CLL, hypogammaglobulinaemia is observed in more than half of patients, often worsening with disease progression (12). In MM, immunoparesis can affect multiple immunoglobulin isotypes, increasing susceptibility to infection (7, 12).Immunosuppressive agents affect humoral immunity via distinct mechanisms (13, 14). Corticosteroids suppress Ig synthesis and lymphocyte proliferation (14); alkylating agents (e.g., cyclophosphamide) cause lymphodepletion (14); antimetabolites (e.g., methotrexate, azathioprine) (14) impair proliferation; and monoclonal antibodies like rituximab (anti-CD20) (15) or daratumumab (anti-CD38) (7, 14) deplete B-cells or plasma cells directly (15–17). CD20 is one of the surface markers of B cells (15) whilst CD38 is a marker of plasma cells (7, 14). Rituximab targets CD20 (16), leading to prolonged B-cell depletion and subsequent hypogammaglobulinaemia (15–17), typically affecting IgM initially, followed by IgG and IgA (15–17). Studies in multiple sclerosis (MS) (15) and neuromyelitis optica spectrum disorder (NMOSD) (15) have found hypogammaglobulinaemia in 12% of treated patients (15), with age over 50, rituximab exposure, and lower baseline immunoglobulin levels identified as key risk factors (15). Similar trends are seen in antineutrophil cytoplasmic antibody (ANCA)-associated vasculitis (AAV) (16), where over 40% of patients developed hypogammaglobulinaemia (16) within six months of rituximab treatment. In these cases, older age and higher cumulative glucocorticoid exposure were the strongest independent predictors (16). Patients with rheumatic diseases, such as rheumatoid arthritis (RA) (17) and systemic lupus erythematosus (SLE) (17), have demonstrated increased risk of hypogammaglobulinaemia and infections (17), particularly those with prior cyclophosphamide exposure (17) or persistent immunosuppressive therapy (17).These observations highlight the importance of thorough risk assessment and baseline testing before initiating B-cell depleting therapy (9, 12). Regular monitoring of immunoglobulin levels and lymphocyte counts during treatment can help identify patients at risk of serious infections (12) and guide adjustments in immunological care (12).

Emerging cancer therapies (13), such as chimeric antigen receptor T-cell (CAR-T) (13) treatments, may contribute to SADs by causing profound B-cell depletion (13). Patients receiving CAR-T therapy targeting CD19 (13) or those undergoing haematopoietic stem cell transplantation (14) often develop hypogammaglobulinaemia due to myeloablative conditioning (14), delayed B-cell recovery, and graft-versus-host disease (14).

### 2.2 Increased immunoglobulin loss

Increased loss of immunoglobulins is another important mechanism underlying SADs (9, 12). Protein-losing enteropathies (9), including coeliac disease (9), inflammatory bowel disease (9, 12), intestinal lymphangiectasia (9, 12), and others (12), can result in substantial enteric loss of immunoglobulins (9), often accompanied by hypoalbuminaemia and lymphopenia (9, 12). This may result from one of three mechanisms: direct mucosal injury (such as in ulcerative conditions) (9), increased permeability of the intestinal lining (9), or loss of lymph due to lymphatic obstruction (in intestinal lymphangiectasia) (9).

Similarly, in nephrotic syndrome and other protein-losing nephropathies, significant urinary loss of IgG can occur (9, 12). These patients typically have intact B-cell function (4) but are more susceptible to recurrent infections (9), especially with encapsulated organisms such as *Streptococcus pneumoniae* and *Haemophilus influenzae (* 4, 9, 12 *)*.

It is difficult to determine the true proportion of patients with hypogammaglobulinaemia in the context of enteropathies or nephropathies, owing to the heterogeneity of underlying conditions (9) and confounding factors such as concomitant immunosuppressive treatment (12), particularly B cell–targeted therapies (15–17), which may independently impair IgG synthesis.

### 2.3 Other causes

Good’s syndrome (18) is a rare but important cause of SADs, associated with thymoma, profound B-cell lymphopenia, and combined humoral and cellular immune defects (18). Although it shares some features with common variable immunodeficiency (CVID), it typically occurs later in life and has a distinct immunological profile (18). The exact mechanisms of Good’s syndrome remain poorly understood (12, 18), and further studies are needed to improve diagnosis and treatment.

Malnutrition (9, 12), especially protein-calorie malnutrition, impairs immunoglobulin synthesis and contributes to secondary immunodeficiency (2, 12). Cachexia in chronic diseases (9, 12) (e.g., advanced cancer, chronic kidney disease) similarly depletes immunoglobulin reserves and lymphocyte populations (2, 12). Severe trauma, burns, and critical illness have been associated with transient hypogammaglobulinaemia (12). However, the clinical significance of this finding remains to be fully established.

Understanding the diverse causes of SADs is critical for informing treatment decisions (9), including the suitability and duration of IgRT (12), the role of antimicrobial prophylaxis, and the potential for reversibility of the immunodeficiency (7, 10, 11).

## 3 Clinical presentation and red flags

The clinical presentation of SADs is varied and often overlaps with primary immunodeficiencies (1–4, 12). Although SADs typically emerges in adults with known underlying conditions, (1–4) such as haematological malignancies, or protein-losing states, increasing use of immunosuppressants in paediatric populations (2, 9) (e.g., for inflammatory bowel disease or nephrotic syndrome) warrants consideration of SADs in children as well (2, 9). The most common and characteristic feature of SADs is recurrent bacterial infections (2), particularly of the respiratory tract (2, 12).

Patients often present with repeated or severe sinopulmonary infections, including otitis media, sinusitis, bronchitis, and pneumonia (12, 19). Infections with encapsulated bacteria such as *Streptococcus pneumoniae* and *Haemophilus influenzae* are particularly common (2–4, 12).Gastrointestinal infections, for example with *Giardia lamblia* or *Campylobacter*, and persistent diarrhoea may also occur (2, 3, 12).In some cases, patients may experience skin and soft tissue infections, most frequently caused by staphylococcal or streptococcal species (1–3).

A number of key clinical indicators should prompt suspicion of antibody deficiency (1, 2, 12, 19):

- Two or more severe infections within a six to twelve-month period requiring intravenous antibiotics or hospital admission (10, 12, 19).
- Opportunistic infections, including *Pneumocystis jirovecii* pneumonia (PJP), particularly in those receiving B-cell depleting therapies (14).
- Persistently poor vaccine responses or waning post-vaccination antibody titres (19).
- Known use of treatments that affect B-cell function, with delayed immune recovery (14).
- Evidence of chronic lung changes or bronchiectasis on imaging studies (19).

Non-infectious complications are less common is SADs in comparison to PADs. Although autoimmune cytopenias are frequently observed in patients with SADs secondary to haematological malignancies or immunosuppressive therapy (2), their presence should also prompt consideration of an underlying PADs, particularly in younger patients or those with other suggestive features of immune dysregulation (2, 9).

Emerging evidence underscores the importance of careful, structured review of clinical data and longitudinal monitoring to identify patients at risk of SADs (12, 19, 20). Regular measurement of immunoglobulin levels (12), review of infection patterns (9), and awareness of treatments such as rituximab that affect B-cell populations (16) are all vital to ensuring timely recognition and optimal management.

## 4 Laboratory assessment of secondary antibody deficiencies

A thorough and systematic laboratory assessment is essential when evaluating a patient with suspected SADs. This process confirms the diagnosis, clarifies the severity of immunoglobulin deficiency, and guides decisions regarding treatment and monitoring. Testing (19) should be tailored to the clinical context (12), underlying condition (16), and infection history, with repeated measurements often necessary in patients receiving immunosuppressive therapy.

### 4.1 Serum immunoglobulin quantification

Measurement of serum IgG, IgA, and IgM remains central to the initial assessment (19) of secondary antibody deficiency. An IgG level below 4 g/L is strongly linked to serious infection risk, particularly in haematological malignancy. IgG subclass deficiencies, especially IgG2, may also impair vaccine responses (12, 19).

Patients with IgG levels between 4–6 g/L and poor vaccine responses require careful evaluation. Decisions regarding immunoglobulin replacement therapy (IgRT) should consider infection burden and functional antibody testing. In selected cases, a trial of antimicrobial prophylaxis may be appropriate before starting IgRT (12).

Re-evaluation is recommended after 6–12 months of IgRT, focusing on infection history (12), IgG trough levels, and any evidence of immune recovery. Discontinuation may be considered if the underlying cause resolves, immunosuppression is reduced, or vaccine responses improve. Ongoing monitoring of IgG levels and antibody function is essential during and after treatment (12). Practices does vary across regions and countries; this has been discussed below (21).

### 4.2 Specific antibody responses

Assessment of specific antibody responses is a critical part of evaluating the functional integrity of humoral immunity (19). Both protein and polysaccharide antigen responses should ideally be assessed (19). Measurement of baseline and post-vaccination antibody titres to tetanus and diphtheria toxoids, as well as to the pneumococcal polysaccharide vaccine (PPV23) or conjugate vaccines (PCV13 or PCV20), is recommended (12, 19). Post-vaccination titres are typically measured 4 to 6 weeks after immunisation to allow adequate time for an antibody response.

An adequate response is generally defined as either (12, 19):

– a fourfold rise from baseline in specific antibody titres, or
– attainment of antibody concentrations above defined protective thresholds (e.g. ≥0.35 µg/mL for at least 50–70% of tested pneumococcal serotypes (12), depending on local laboratory standards).

Failure to mount or maintain these responses strongly suggests a functional antibody defect (19), even if total immunoglobulin levels are within the normal range (5). However, given the increasing use of PCV20 in many immunisation programmes (12), interpretation of responses has become more nuanced, as coverage of individual serotypes may vary. In children under two years of age, responses to polysaccharide vaccines such as PPV23 are unreliable due to immature T-cell–independent immunity; conjugate vaccines are therefore preferred for assessment in this age group. Practices does vary across regions and countries.

### 4.3 Serum protein electrophoresis and immunofixation

These tests help to distinguish polyclonal hypogammaglobulinaemia, as seen in SADs, from paraproteinaemia or monoclonal gammopathies, which can present with similar laboratory features. Serum free light chain analysis and assessment of the kappa/lambda ratio can provide further insights in suspected cases of monoclonal gammopathy (1, 8). In addition to aiding in the diagnosis of monoclonal gammopathies, low levels of serum free light chains (sFLCs) and lack of recovery post-therapy have recently been proposed as potential markers of persistent antibody deficiency (15) in patients with haematological malignancy or prolonged B-cell depletion (16). Further prospective studies are warranted to validate their utility in routine immunological assessment.

### 4.4 Lymphocyte subset analysis by flow cytometry

Quantification of CD19+ B cells, CD3+ T cells, and CD16/56+ NK cells using flow cytometry is valuable in identifying B-cell depletion or broader immune defects (19). This is particularly relevant for patients who have received rituximab or CAR-T cell therapy (13, 16), or for those suspected of having Good’s syndrome (18), where there is often a profound absence of circulating B cells (18), as well as uncovering an underlying primary immunodeficiency in patients with recurrent infections (19) and persistent hypogammaglobulinaemia initially attributed to SADs.

Good’s syndrome (18, 20), CVID (20), and SADs (20–24) all involve low immunoglobulin levels and recurrent infections (20, 24) but differ in their causes and immune profiles (24). Good’s syndrome (18) is linked to thymoma and features combined immunodeficiency (18), with absent B cells and T-cell defects. CVID is a primary antibody deficiency with normal or low B-cell counts and impaired antibody production (24); T-cell abnormalities may be present but are usually milder (20, 24). SADs are acquired, often due to malignancy, immunosuppressive treatment, or protein loss (1–4). In SADs, B- and T-cell numbers are usually intact unless affected by therapy (13, 14, 17). Unlike the primary forms, SADs may improve if the underlying cause is addressed.

### 4.5 Additional investigations

Additional laboratory tests are often helpful to identify secondary causes of hypogammaglobulinaemia (20–24). also, these may differ from region/country to an other (20–24); depends upon the local guidelines, consensus or practice (24).

Investigations may include serum albumin and urinary protein quantification to assess for nephrotic-range proteinuria (22), and faecal alpha-1-antitrypsin to evaluate for protein-losing enteropathy (12, 22). Imaging studies such as high-resolution chest CT can reveal structural lung disease or bronchiectasis in patients with recurrent pneumonia (12).

Interpreting laboratory data in SADs requires careful consideration of the clinical picture (25), treatment history, and potential reversibility of the immune defect (20, 24). Repeated testing may be needed during periods of immunosuppression or in patients recovering from chemotherapy (22). It is important to remember that not all patients with low immunoglobulin levels and low vaccination response will require immunoglobulin replacement therapy (26); decisions should be guided by infection risk and the presence of functional antibody defects (25, 26).

Emerging data from structured clinical assessments support the use of longitudinal laboratory monitoring, alongside careful review of infection history and immunosuppressive treatments, to identify those most likely to benefit from early intervention (27).

## 5 Management strategies: immunoglobulin replacement and beyond

Management of secondary antibody deficiencies (SADs) requires an individualised, patient-centred approach that considers the underlying cause, the severity of immunoglobulin deficiency, and the clinical impact on the patient. Strategies typically involve immunoglobulin replacement therapy (IgRT), antimicrobial prophylaxis, vaccination, and, where possible, modification or withdrawal of immunosuppressive treatments.

### 5.1 Indications for immunoglobulin replacement therapy

IgRT is generally considered in patients with documented hypogammaglobulinaemia (typically IgG <4 g/L) and recurrent or severe infections (26). However, serum IgG levels alone are not sufficient to guide treatment decisions. Functional assessment of specific antibody responses to vaccines is critical for identifying those with clinically relevant humoral immune impairment (26); in addition to regional variation in guidelines and practices (23, 28–35) in using IgRT.

Evidence increasingly highlights the significant risk of hypogammaglobulinaemia as a complication of B-cell-depleting therapies (35), such as rituximab and ocrelizumab (15–17), used in autoimmune neurological disorders (15), connective tissue diseases (17), systemic vasculitis (16), CLL (21), post-transplant states (24), and other conditions (22). Studies in multiple sclerosis (MS) (15) and neuromyelitis optica spectrum disorder (NMOSD) (15) have found hypogammaglobulinaemia in 12% of treated patients (15), with age over 50, rituximab exposure, and lower baseline immunoglobulin levels identified as key risk factors (15). Similar trends are seen in antineutrophil cytoplasmic antibody (ANCA)-associated vasculitis (AAV) (16), where over 40% of patients developed hypogammaglobulinaemia (16) within six months of rituximab treatment. In these cases, older age and higher cumulative glucocorticoid exposure were the strongest independent predictors (16). Patients with rheumatic diseases, such as rheumatoid arthritis (RA) (17) and systemic lupus erythematosus (SLE) (17), have demonstrated increased risk of hypogammaglobulinaemia and infections (17), particularly those with prior cyclophosphamide exposure (17) or persistent immunosuppressive therapy (17). Regular monitoring of immunoglobulin levels and lymphocyte counts during treatment can help identify patients at risk of serious infections (12) and guide adjustments in immunological care (12).

Identifying at risk population early (27) would reduce long term complications (27). Structured review of infection patterns and laboratory data from electronic health records is being used to identify those at greatest risk, helping to ensure more timely and appropriate use of IgRT (27).

### 5.2 Route of administration and dosing

IgRT can be administered either intravenously (6, 29) (IVIG) or subcutaneously (6, 29) (SCIG). IVIG is usually given every three to four weeks (6, 29) and can rapidly raise serum IgG levels, but it requires hospital-based infusion and carries a higher risk of systemic reactions (6). SCIG is often preferred by many patients for its convenience (6), steady serum IgG concentrations, and lower incidence of systemic side effects (20, 29). Initial dosing is typically 400–600 mg/kg per month, adjusted based on infection frequency and trough IgG levels, aiming for levels above 6–8 g/L (3, 29).

### 5.3 Adjunctive measures and supportive strategies

Antimicrobial prophylaxis is valuable for patients with milder SADs (26) who are not candidates for IgRT (26), or during periods of heightened infection risk, such as neutropenia. Co-trimoxazole and azithromycin are frequently used (4, 26). Co-trimoxazole is often favoured in patients at risk of *Pneumocystis jirovecii* pneumonia or recurrent urinary tract infections, while azithromycin is commonly prescribed for respiratory tract prophylaxis or for its anti-inflammatory benefit (12, 20, 26).

Vaccination remains a key preventive measure in many patients with suspected SADs, such as, pneumococcal, influenza, and *Haemophilus influenzae* type b, are recommended (12, 25); however, the practice is different across regions and countries (28–35), **Table 1** summarise some of these main variations in practice. Whereas live vaccines are generally avoided in those with suspected immunodeficiency or on immunosuppressive therapy. However, vaccine recommendations should be tailored to the patient’s risk profile and guided by expert advice, in line with the regional guidelones and recommendations (28–35).

**Table 1:** Guideline comparison of vaccination, vaccine-response assessment, antibiotic prophylaxis and immunoglobulin replacement in secondary immunodeficiency: summary of recent international recommendations.

Source (short)	Vaccination practice	Act on vaccine response	Antibiotic prophylaxis	Ig replacement therapy (IgRT)	Notes
BSI/UKPIN 2022 (6) – Consensus (general IgRT practice)	Adhere to standard immunisation strategies; not the primary focus.	Assess immune recovery and clinical control over time.	Contextual—part of broader infection prevention strategy.	If stable and infection-free on IgRT, consider dose reduction/cessation with close review (humoral recovery).	UK consensus emphasising review for de-escalation when stable.
NCCP (Ireland) 2021/2023 (30) – Patient selection for IgRT (SID)	Incorporates assessment of response to polysaccharide vaccine challenge when appropriate.	Document failure of antibody response to unconjugated pneumococcal (or other polysaccharide) challenge when judging IgRT eligibility.	Trial of continuous oral prophylaxis for ~6 months before IgRT (except a single life-threatening infection).	Consider when: recurrent/severe infections despite ≥6-month prophylaxis OR one life-threatening infection; plus underlying B-cell malignancy/therapy-related hypogammaglobulinaemia; AND IgG <4 g/L (exclude paraprotein); AND/OR failed polysaccharide response; clinician judgement applies. Dosing 0.4–0.6 g/kg/month; monitor troughs; reduce/stop if recovery.	Provides explicit ‘ladder’ and thresholds; includes post-CAR-T notes.
European expert consensus 2021 (5) – SADs in haematological malignancies	Optimise routine vaccinations per general guidance; use ‘test immunisation’ to assess function when needed.	Post-immunisation specific antibody measurement (typically to polysaccharide antigens) can guide decisions for IgRT in borderline cases.	Escalate to IgRT when infections persist despite appropriate anti-infectives.	Recommend IgRT for severe/recurrent/persistent infections despite anti-infectives; supports SCIg; provides dosing/stop principles (individualised).	Consensus statements covering initiation, dosing, discontinuation.
ESGICH 2018 (32) – Targeted/biologic therapies (Infectious diseases perspectives)	Favors inactivated vaccines; advise vaccination before starting targeted/biologic agents where feasible; avoid live-attenuated under significant immunosuppression.	Not a primary focus; general risk appraisal.	Agent-specific prophylaxis and surveillance should be considered based on infection risks of therapies (e.g., B-cell–depleting).	Not central to this document; may be considered in selected SID settings.	Framework for infection prevention around targeted/biologic agents.
EULAR 2019 (36)– Vaccination in AIIRD (adults)	Annual influenza; sequential pneumococcal (conjugate→polysaccharide); risk-based HBV, HPV, zoster. Inactivated vaccines are safe; live-attenuated may be considered with caution; vaccinate before or away from intense immunosuppression (esp. B-cell depletion).	Check serology when clinically relevant (e.g., HBV) to document protection.	Not addressed.	Not addressed.	Use as the baseline vaccination schedule to align other SID guidance to.
ESGICH 2018 (32) – Targeted/biologic therapies (Infectious diseases perspectives)	Favors inactivated vaccines; advise vaccination before starting targeted/biologic agents where feasible; avoid live-attenuated under significant immunosuppression.	Not a primary focus; general risk appraisal.	Agent-specific prophylaxis and surveillance should be considered based on infection risks of therapies (e.g., B-cell–depleting).	Not central to this document; may be considered in selected SID settings.	Framework for infection prevention around targeted/biologic agents.
Clinician Survey in Europe 2023 (37)	Reinforces standard vaccinations; ideally pre-immunosuppression.	Poor polysaccharide responses + infection burden support IgRT.	Recommends stepwise approach—vaccines → prophylactic antibiotics → IgRT for failures.	Practical dose/route considerations (IVIG/SCIG) and monitoring/stop strategies.	Real-world, Q&A format to operationalise guidance.
Spanish consensus 2023 (31) – SID in haem malignancy	Active immunisation against seasonal influenza (incl. H1N1), pneumococcus, and Haemophilus influenzae recommended.	Monitor IgG every ~3 months; protocolised IVIG use with trough monitoring; consider stopping IVIG after ~12 months if IgG recovery and infections controlled.	Part of prevention bundle (practice-based).	Endorses having a protocol for IVIG use in SID; monitor troughs to titrate; stop IVIG when recovery documented.	Delphi-style expert consensus; pragmatic monitoring cadence.
Middle East SID Delphi consensus (33)	Positions vaccination as a core prevention pillar within a ‘bundle’ (with access to rescue antibiotics and prophylactic antibiotics). No specific schedule—follow regional/national guidance.	Not prescriptive; evaluate humoral immunity as part of SID work-up when deciding on IgRT.	Explicitly includes preventive antibiotics and rapid access to empiric antibiotics as part of standard care.	Consider for severe/recurrent infections despite vaccination + prophylactic antibiotics in patients with low IgG; notes EMA expansion of IgRT indications beyond CLL/MM (e.g., B-cell–depleting therapy, post-transplant).	Region-specific consensus; emphasises stepwise prevention bundle.
Clinician Survey in China 2021 (34)	Reported under-use/heterogeneity of vaccination in SID care.	Variable use of test immunisation/serology in practice.	Oral prophylaxis used variably; often before/with IgRT.	Starting doses and target troughs varied; highlights practice gaps vs guidance.	Describes real-world variation; supports need for standardised pathways.

Where possible, the underlying cause of SAD should be addressed. Reduction or withdrawal of immunosuppressive therapy has led to partial or complete recovery of immunoglobulin levels in some cases, particularly after discontinuation of rituximab or tapering of corticosteroids (35).

### 5.4 Monitoring, re-evaluation and regional variations

Ongoing assessment is essential to ensure the effectiveness and safety of IgRT and to identify any new complications (12, 26, 35). Follow-up should include (12):

- Monitoring infection frequency, severity, and antibiotic use.
- Measurement of serum trough IgG levels to guide dose adjustments.
- Evaluation of adverse effects, including thromboembolic events, haemolysis, or renal impairment in high-risk patients.
- Periodic reassessment of the need for IgRT, particularly in patients with potentially reversible immunodeficiency.

Structured evaluation of clinical data, including infection history, laboratory markers, and immunosuppressive exposure, is increasingly used to identify patients at risk of SADs and to ensure early, targeted interventions (12).

A multidisciplinary (12), structured approach involving clinical immunology specialists (20) is essential for optimal management. Detailed review of history and laboratory results, careful monitoring, and tailored care plans reduce infection risk, improve outcomes, and promote consistent, evidence-based practice across healthcare settings (12, 20, 35). Patients with SADs should ideally be managed in partnership with immunology services to ensure accurate diagnosis and long-term follow-up (12, 20).

As described in **Table 1**, the management of SADs across a range of underlying conditions; including haematological malignancies, autoimmune disorders, and other immunosuppressive states in different regions show broad agreement on core principles, yet practice remains shaped by regional priorities, healthcare infrastructure, and specialist perspectives.

## 6 Future directions and research priorities

The field of SADs is evolving rapidly, driven by the challenges of increasing therapeutic complexity, expanding at-risk populations, and the growing integration of data-driven approaches in healthcare. Improving recognition, management, and outcomes for patients with SAD will require coordinated progress in clinical research, laboratory innovation, and collaborative, multidisciplinary care.

### 6.1 Personalised risk stratification

Traditional approaches to SAD have largely focused on fixed IgG thresholds and the frequency of infections. However, these criteria may not adequately reflect the individual risk of complications in many patients. Future strategies should aim to incorporate more detailed risk stratification tools, including IgG subclass deficiencies, vaccine response profiles, and cumulative immunosuppressive exposure (3, 4). Large-scale registries and prospective cohort studies will be essential to refining these tools and identifying reliable predictors of infection risk and treatment response (27, 38).

### 6.2 Role of structured clinical data, AI models, and health records

The analysis of structured clinical data, such as longitudinal laboratory trends and treatment histories, is increasingly recognised as an important tool for the early identification of SAD (38). Drawing lessons from emerging models in primary immunodeficiency, advanced algorithms and large-scale deep learning models have demonstrated the ability to identify at-risk individuals using patterns within electronic health records (EHRs) (30, 40). These AI-driven models have achieved high accuracy in predicting undiagnosed immunodeficiency, highlighting the potential of similar strategies to improve early diagnosis of SAD.

**Figure 1:**
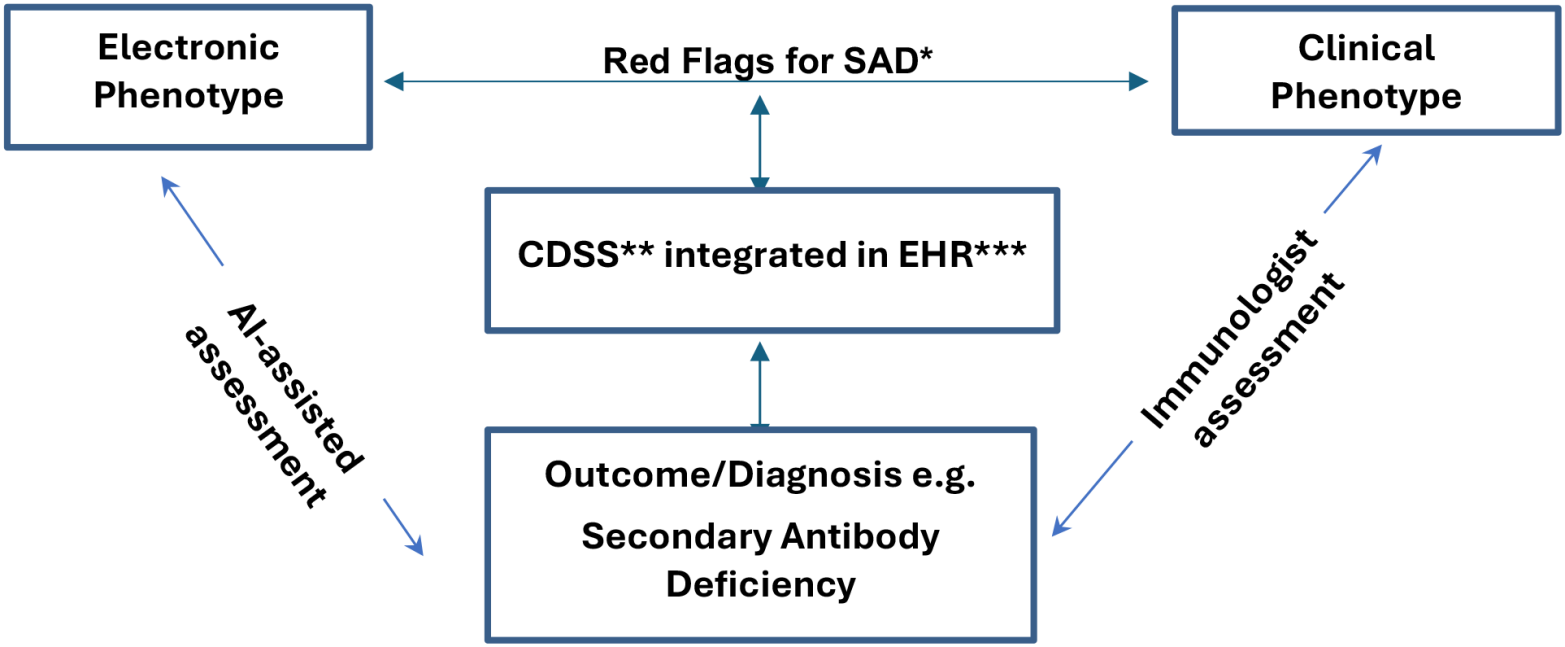
Roles of Immunologist assisted by AI in designing, assessing, and facilitating the early diagnosis of Secondary Antibody Deficiency when appropriate through clinical decision support systems. SAD*, Secondary Antibody Deficiency; CDSS**, Clinical Decision-Support System; EHR***, Electronic Health Record.

Explainable AI (XAI) frameworks have further strengthened these approaches, offering insights into decision-making processes and increasing trust among clinicians and patients (42). There is also growing interest in large language models and multimodal AI systems, which integrate diverse clinical data, imaging, and laboratory findings to provide a more comprehensive understanding of patient risk (39, 41). The incorporation of these models into clinical decision support systems could transform the identification and management of SAD, enabling more precise and timely interventions for at-risk patients. **Figure 1**. A conceptual framework developed by the authors, showing how AI-assisted clinical decision support systems (CDSS) integrated with electronic health records (EHRs) can assist immunologists in the early detection and personalised management of SADs.

### 6.3 Expanding the evidence base for immunoglobulin replacement therapy

Much of the current evidence for IgRT in SAD comes from studies involving haematological malignancies and post-transplant immunodeficiency (27, 39). There remains a clear need to extend this evidence base to include patients with other underlying conditions, such as protein-losing enteropathies, autoimmune diseases, and those receiving long-term immunosuppressive therapy outside of oncology. Future studies should adopt standardised outcome measures, including infection-related hospitalisation rates, quality-of-life assessments, and antimicrobial usage, to understand the broader benefits and long-term safety of IgRT in these populations (27, 42).

### 6.4 Biomarker development, multi-omics approaches, and laboratory advances

Laboratory testing is already central to the diagnosis and monitoring of SAD, with assessments such as serum free light chains, lymphocyte subset enumeration, and vaccine response profiling offering essential information (3, 12, 20, 42). Advances in multi-omics technologies; including transcriptomics, proteomics, and metabolomics; are now providing deeper insights into immune recovery patterns and potential biomarkers for stratifying infection risk and predicting IgRT response (43, 44). Future research should integrate these data-rich approaches with clinical outcomes, enabling more precise management and avoiding unnecessary treatment in lower-risk patients.

### 6.5 Collaborative and multidisciplinary approaches

A multidisciplinary approach will continue to be essential to optimise patient outcomes. Collaboration between haematology, rheumatology, neurology, and clinical immunology is critical to ensure timely diagnosis, accurate laboratory interpretation, and consistent access to specialist care. Building on lessons from the identification of primary immunodeficiency within B-cell lymphoproliferative disorders (27, 39, 40), these collaborations will help to ensure equitable, evidence-based care for all patients with SAD.

As the field advances, there is a pressing need to harmonise diagnostic criteria, treatment thresholds, and laboratory assessments across healthcare systems. The integration of AI-driven models, health record analysis, and multi-omics data promises to transform the early detection and management of SAD, ensuring that patients receive the best possible care guided by both clinical expertise and cutting-edge technologies.

## 7 Conclusion

Secondary antibody deficiencies are common, clinically significant, and frequently under-recognised. Timely diagnosis, structured laboratory evaluation, and appropriate use of immunoglobulin replacement or supportive strategies can reduce infection burden and improve outcomes. Greater consistency in diagnostic thresholds and treatment criteria, supported by closer collaboration between specialties, will be essential. Digital tools and large-scale data analysis hold promise for earlier recognition, but clinical expertise and equitable access to immunology services remain central to effective care.
